# Prognostic Nutritional Index, Circulating Bacterial and Viral DNA, and Neurocognitive Vulnerability in Geriatric Inpatients: A Cross-Sectional Study

**DOI:** 10.3390/nu18203387

**Published:** 2026-10-15

**Authors:** Gretta Veronica Badillo Pazmay, Patrizia D’Aquila, Carlo Fortunato, Laura Cianfruglia, Dolores Limongi, Carla Prezioso, Federica Novazzi, Pietro Giorgio Spezia, Marta Balietti, Marco Malavolta, Francesco Piacenza, Maurizio Cardelli, Tiziana Casoli, Rosanna Maniscalco, Francesca Marchegiani, Rina Recchioni, Giulia Matacchione, Chiara Giordani, Matilde Sbriscia, Sonia Fantone, Mirko Di Rosa, Antonio Cherubini, Leonardo Biscetti, Giuseppe Pelliccioni, Fabrizia Lattanzio, Anna Rita Bonfigli, Giuseppe Passarino, Fabiola Olivieri, Fabrizio Maggi, Dina Bellizzi, Robertina Giacconi

**Affiliations:** 1 Center of Biogerontology, IRCCS INRCA, 60121 Ancona, Italy; grettaveronica.badillopazmay@unicam.it (G.V.B.P.); c.fortunato2@inrca.it (C.F.); l.cianfruglia.inrca@gmail.com (L.C.); m.balietti@inrca.it (M.B.); m.malavolta@inrca.it (M.M.); f.piacenza@inrca.it (F.P.); m.cardelli@inrca.it (M.C.); t.casoli@inrca.it (T.C.); r.maniscalco@inrca.it (R.M.); m.sbriscia@inrca.it (M.S.); f.olivieri@inrca.it (F.O.); 2 Department of Biology, Ecology and Earth Sciences, University of Calabria, 87036 Rende, Italy; patrizia.daquila@unical.it (P.D.); giuseppe.passarino@unical.it (G.P.); dina.bellizzi@unical.it (D.B.); 3 Department for the Promotion of Human Sciences and Quality of Life, San Raffaele University, 00166 Rome, Italy; dolores.limongi@sanraffaele.it (D.L.); carla.prezioso@uniroma5.it (C.P.); 4 Laboratory of Microbiology, IRCCS San Raffaele Roma, 00166 Rome, Italy; 5 Department of Medicine and Technological Innovation, University of Insubria, 21100 Varese, Italy; federica.novazzi@asst-settelaghi.it; 6 Laboratory of Microbiology, ASST SetteLaghi, 21100 Varese, Italy; 7 Laboratory of Virology and Laboratories of Biosafety, National Institute for Infectious Diseases, Lazzaro Spallanzani-IRCCS, 00149 Rome, Italy; pietro.spezia@inmi.it (P.G.S.); fabrizio.maggi@inmi.it (F.M.); 8 Department of Clinical and Molecular Sciences (DISCLIMO), Università Politecnica delle Marche, 60126 Ancona, Italy; a.cherubini@inrca.it; 9 Clinic of Laboratory and Precision Medicine, IRCCS INRCA, 60121 Ancona, Italy; f.marchegiani2@inrca.it (F.M.); r.recchioni@inrca.it (R.R.); g.matacchione@inrca.it (G.M.); c.giordani@inrca.it (C.G.); 10 Geriatrics and Clinical Epidemiology, IRCCS INRCA, 87100 Cosenza, Italy; m.dirosa@inrca.it; 11 Geriatria, Accettazione Geriatrica e Centro di Ricerca per l’Invecchiamento, IRCCS INRCA, 60121 Ancona, Italy; 12 Neurology Unit, IRCCS INRCA, 60124 Ancona, Italy; l.biscetti@inrca.it (L.B.); g.pelliccioni@inrca.it (G.P.); 13 Scientific Direction, IRCCS INRCA, 60127 Ancona, Italy; f.lattanzio@inrca.it (F.L.); a.bonfigli@inrca.it (A.R.B.)

**Keywords:** immunonutritional status, gut-brain axis, hospitalized older adults, blood bacterial DNA, virome, neurocognitive and behavioral conditions, cognitive impairment, inflammaging, telomeres

## Abstract

Background/Objectives: Impaired immunonutritional status is common among hospitalized older adults and may contribute to adverse outcomes through alterations in gut–brain axis pathways involving immune, microbial, and inflammatory mechanisms. The Prognostic Nutritional Index (PNI), a composite marker based on serum albumin and lymphocyte count, may identify patients with altered immunonutritional status and increased clinical vulnerability. This cross-sectional study investigated whether PNI-defined immunonutritional impairment is associated with bacterial and viral DNAemia, inflammation, and neurocognitive conditions in 1016 geriatric patients from the Report-AGE cohort. Methods: Participants were stratified according to PNI into low- and high-PNI groups. Blood bacterial DNA (BB-DNA), Torque teno virus (TTV), herpes simplex virus (HSV-1), varicella-zoster virus (VZV), leukocyte telomere length (LTL), inflammatory and neurodegeneration-related biomarkers were evaluated. Multivariable logistic regression models were used to assess the associations of BB-DNA and PNI with cognitive impairment and interRAI-derived neurocognitive conditions. Results: Low-PNI patients exhibited a higher burden of comorbidities, frailty, and systemic inflammation, alongside increased concentrations of BB-DNA, IL-6, IL-17A, GDF15, osteopontin, FGF21, follistatin, and neurofilament light chain. Among patients with low-PNI, increasing BB-DNA tertiles were associated with progressively higher TTV, HSV-1, and VZV DNAemia, elevated NLR, IL-6, and IL-17A levels, and shorter LTL. In multivariable analyses, low-PNI was independently associated with cognitive impairment (OR: 1.39, 95% CI: 1.06–1.83), easily distracted behavior (OR: 1.75, 95% CI: 1.29–2.35), and behavioral disturbances (OR: 1.80, 95% CI: 1.05–3.06), whereas BB-DNA showed an association with acute changes in mental status (OR: 1.75, 95% CI: 1.09–2.81) and behavioral disturbances (OR: 3.09, 95% CI: 1.11–8.59). Conclusions: These findings suggest that impaired immunonutritional status, bacterial and viral burden, systemic inflammation, and neurocognitive alterations may represent interconnected features of clinical vulnerability, although the cross-sectional design does not establish temporal or causal relationships among these factors.

## 1. Introduction

Malnutrition is highly prevalent among hospitalized older adults, with estimates suggesting that it affects up to 30% of individuals aged ≥65 years. It is associated with adverse outcomes, including prolonged hospital stay, increased in-hospital mortality, and higher risk of post-discharge institutionalization [1,2]. Beyond the depletion of nutritional reserves, malnutrition impairs immune competence and may contribute to inflammaging [3]. Persistent elevation of pro-inflammatory mediators and sustained immune dysregulation contribute to frailty, multimorbidity, neurodegeneration, and mortality in late life [4,5,6]. Nutritional impairment may therefore contribute to increased brain vulnerability in older adults, being associated with acute cognitive dysfunction, including delirium, and potentially with longer-term neurodegenerative trajectories [7,8,9].

These pathways may converge on the gut–brain axis. Age-related dysbiosis and progressive loss of intestinal barrier integrity can promote microbial translocation, including blood bacterial DNA (BB-DNA) and endotoxins, into the systemic circulation [10,11]. In older individuals, BB-DNA has been linked to alterations in intestinal barrier integrity, systemic inflammation, immune activation, and biological aging, and may represent a circulating marker of microbial translocation and barrier dysfunction [10,12,13,14,15]. Through toll-like receptor (TLR) signaling, microbial translocation may sustain cytokine release, blood–brain barrier disruption, and neuroinflammation [16]. Impaired immune surveillance may favor latent viral reactivation. Herpes simplex virus type 1 (HSV-1) and varicella-zoster virus (VZV) frequently reactivate in frail and malnourished individuals [17,18,19]. Torque teno virus (TTV), a ubiquitous non-pathogenic anellovirus, has been proposed as an indirect marker of immune competence, as higher viremia reflects reduced immune control and impaired antiviral surveillance [20]. High TTV loads have been associated with immunosenescence, frailty, and mortality in older adults [21,22,23].

The Prognostic Nutritional Index (PNI) represents a composite measure of nutritional and immunological status based on serum albumin concentration and lymphocyte count [24,25]. Serum albumin reflects protein nutritional status and hepatic synthetic function, whereas lymphocyte count serves as a marker of cell-mediated immunity, which is closely linked to nutritional status [26]. Beyond its original application in oncology and surgical settings [27,28], PNI has been increasingly investigated in older adults and in chronic disease populations as a marker associated with nutritional risk, frailty, systemic inflammation, and adverse clinical outcomes [24,25,29,30,31]. However, whether poor immunonutritional status identifies a broader immune–barrier phenotype associated with microbial and viral burden, inflammatory alterations, cellular aging, and neurocognitive vulnerability in hospitalized older adults remains poorly defined.

In this study, our main research question was whether PNI-defined immunonutritional impairment is associated with circulating bacterial DNA and neurocognitive vulnerability in hospitalized older adults. We additionally examined viral DNAemia within the primary analytical framework to characterize the relationship between viral burden and circulating bacterial DNA across PNI strata. Inflammatory and stress-related biomarkers, leukocyte telomere length, as well as domain-specific neurocognitive measures, were examined in additional exploratory analyses to further characterize the biological and clinical context of these associations. Given the cross-sectional design, these analyses were intended to identify patterns of association rather than establish temporal or causal relationships among immunonutritional status, microbial and viral burden, inflammation, cellular aging, and neurocognitive outcomes. We hypothesized that lower PNI would be associated with higher circulating bacterial DNA and viral DNAemia, altered inflammatory and cellular aging profiles, and greater neurocognitive vulnerability.

## 2. Materials and Methods

### 2.1. Study Cohort Data and Blood Collection

This cross-sectional study was conducted within the framework of the PROMOTERA study (grant no. Grant Number GR-2019-12368606; ethics approval no. CE INRCA 20031, 4 February 2021), which included participants selected from the Report-AGE project a large-scale observational study involving older adults hospitalized at the Italian National Institute of Health and Science on Aging (Istituto Nazionale di Riposo e Cura per Anziani, INRCA-IRCCS) (Trial Registration No. NCT01397682; Ethics Committee No CE INRCA 20031, 4 February 2021) between July 2011 and October 2021 [32]. Participants were eligible if they were 65 years of age or older, had been hospitalized for at least 24 h, and had provided written informed consent. Patients with hematological malignancies or active COVID-19 infection were not considered eligible. Individuals who died during hospitalization were excluded from the present analysis because the study focused on participants who completed the admission assessment and had available biological samples for the planned analyses. The present investigation represents a biological-sample-based substudy of the Report-AGE cohort. Participants were considered eligible for the present analyses on the basis of the availability of stored biological samples and the clinical and laboratory data required for the study. After application of the study-specific eligibility criteria and exclusion of participants who died during hospitalization and those with missing data required for the analyses, the final analytical cohort comprised 1016 participants.

**Figure A1:**
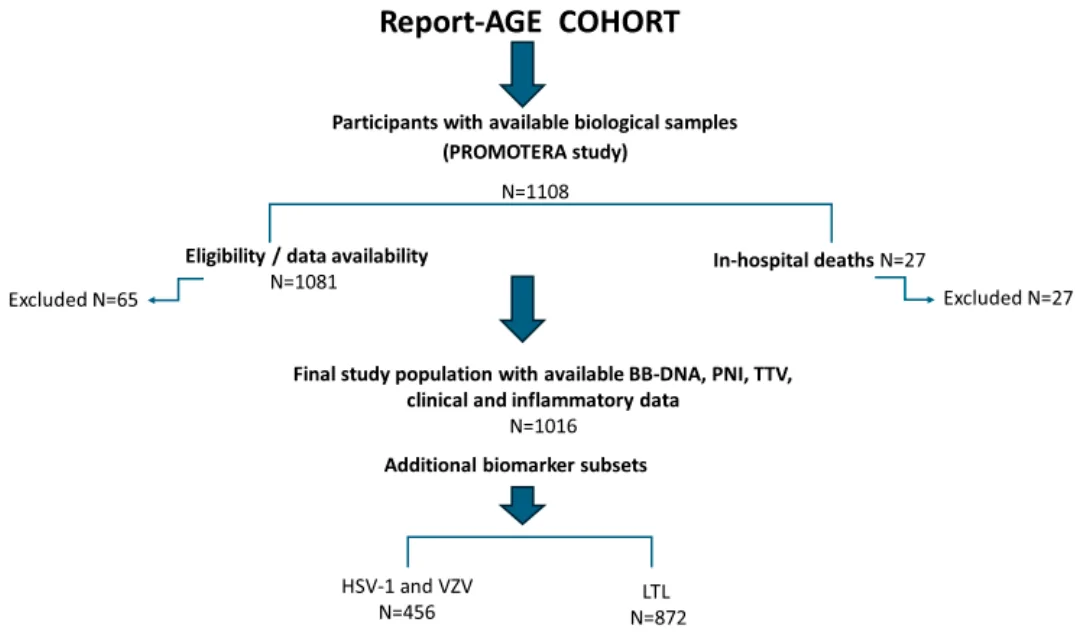
Flowchart of participant selection and derivation of the analytical cohort and biomarker-specific subsets in the Report-AGE study.

In the 1016-participant analytical cohort, BB-DNA, TTV viremia, inflammatory biomarkers, and the clinical variables required for the primary analyses were available. Additional biomarker assessments were available in subsets of the study population: HSV-1 and VZV DNAemia were assessed in 456 participants included in the VIROMA biomarker substudy, for whom the biological material required for herpesvirus characterization was available, whereas LTL was assessed in 872 participants. Thus, HSV-1/VZV and LTL assessments were performed in the corresponding biomarker-specific subsets, whereas evaluations of BB-DNA, TTV, inflammatory biomarkers, and the main clinical variables were conducted in the full analytical cohort of 1016 participants (Figure A1). A comprehensive geriatric assessment (CGA) was performed at admission and discharge using the InterRAI Minimum Data Set for Acute Care (MDS-AC). Blood samples were collected within 24 h of hospital admission in EDTA tubes (Becton, Dickinson and Company, Franklin Lakes, NJ, USA) and stored in the INRCA BioGer Biobank (Ancona, Italy). Routine laboratory testing at admission included blood cell counts, creatinine, albumin, C-reactive protein, and hemoglobin, all determined using standardized laboratory procedures. The glomerular filtration rate (GFR) was estimated using the Chronic Kidney Disease Epidemiology Collaboration (CKD-EPI) equation [33]. The Prognostic Nutritional Index (PNI) was calculated from admission blood samples using the following formula, as previously described [24,34,35]: PNI = 10 × serum albumin value + 0.005 × total lymphocyte count in peripheral blood (mm^3^). PNI values were categorized according to previously proposed PNI thresholds: >50, 45–50, 40–45, and <40 [36]. These categories were used as PNI-defined risk strata and were not considered equivalent to a clinical diagnosis of nutritional status or malnutrition. For the main analyses, participants were categorized into two groups according to PNI: low-PNI (PNI < 40) and high-PNI (PNI ≥ 40). The PNI < 40 threshold was selected to identify participants in the lowest PNI category, representing marked PNI-defined immunonutritional impairment, whereas the PNI ≥ 40 group served as the reference category. Thus, this categorization was intended to contrast participants with markedly low PNI with those above this threshold, rather than to distinguish clinically malnourished from adequately nourished individuals. Comorbidity data were obtained from medical records and classified using the International Classification of Diseases, Ninth Revision (ICD-9). The following conditions were considered: acute myocardial infarction (410–414), congestive heart failure (428), cerebrovascular disease (430–438), dementia (290, 331, 294), depression (296), chronic obstructive pulmonary disease (491, 492, 494, 496), Parkinson’s disease (332), hypertension (401–405), chronic kidney disease (585, 586, 587), anemia (280–285, 790), diabetes (250.0–250.7), and cancer (140–172, 174–195, 200–208). For the purposes of this study, the dementia category also encompassed individuals with mild-to-moderate cognitive impairment who retained decisional capacity and were able to provide informed consent.

Neurocognitive and behavioral conditions were assessed at hospital admission using items from the interRAI Minimum Data Set for Acute Care (MDS-AC). Global cognitive status was evaluated with the Cognitive Performance Scale (CPS), a 7-point multidimensional tool derived from the Minimum Data Set (MDS). The CPS integrates information on memory impairment, level of consciousness, and executive function. Scores range from 0 to 6, where a score ≥ 2 is considered indicative of cognitive impairment, and a score > 4 identifies severe dementia [37]. In addition, specific neurocognitive and behavioral items routinely collected within the interRAI assessment, performed within 24 h of hospital admission, were analyzed individually. These included acute change in mental status from baseline (ACMS), behavioral disturbances (BD), mental function varies over the course of the day (MFV), episodes of disorganised speech (EDS), and easily distracted (ED). ACMS was scored as absent (0) or present (1). MFV, EDS, and ED were scored as 0 (behavior not present), 1 (behavior present but consistent with usual functioning), or 2 (behavior present and different from usual functioning). For the present analyses, these items were dichotomized as absent (0) versus present (1 or 2), whereas behavioral disturbances and ACMS were analysed as binary variables according to the interRAI coding.

Cognitive status was assessed within 24 h of hospital admission. Beyond the global CPS, specific baseline memory domains were evaluated using standardized interRAI MDS-AC items. Clinicians assessed three distinct dimensions via clinical observation and structured tasks, each dichotomized as preserved or impaired according to manual criteria: (i) short-term memory (ability to recall recent information or events within 5 min); (ii) situational memory (orientation to the immediate hospital context and surroundings); and (iii) procedural memory (retention of automated long-term skills and multi-step daily routines, e.g., dressing or eating).

Multimorbidity was evaluated using the Charlson Comorbidity Index (CCI). Frailty was estimated using a Frailty Index according to the deficit accumulation approach by means of a 30-item Frailty Index (FI), incorporating deficits related to comorbidities, functional status, cognition, sensory function, and medication use. The FI was expressed on a continuous scale from 0, indicating no identified deficits, to 1, representing the maximum deficit burden. Based on predefined cut-offs, participants were classified as non-frail (FI < 0.10), pre-frail (0.10–0.19), or frail (FI ≥ 0.20) [38]. The study protocol complied with the principles of the Declaration of Helsinki and received approval from the Ethics Committee of the IRCCS INRCA. Written informed consent was obtained from all participants before study enrollment. The study was reported in accordance with the Strengthening the Reporting of Observational Studies in Epidemiology (STROBE) guidelines for cross-sectional studies [39]. The completed STROBE checklist, including the corresponding page and line numbers in the manuscript, is provided as a separate Supplementary STROBE checklist.

### 2.2. TTV, HSV-1, and VZV DNA Detection and Quantification

Viral DNA was isolated from whole-blood specimens with the QIAamp DNA Blood Mini Kit (Qiagen GmbH, Germany), according to the manufacturer’s instructions. TTV DNA was quantified using a single-step, in-house TaqMan real-time PCR assay, as previously described [40].

This assay targets a highly conserved region within the untranslated region (UTR) of the viral TTV genome using specific forward and reverse primers, enabling quantification of total TTV DNA load without discriminating between individual TTV species coexisting in the same sample.

Detailed procedures for TTV DNA quantification, including assessments of assay specificity, sensitivity, intra- and inter-assay precision, and reproducibility, have been described elsewhere [40]. Briefly, the assay amplifies a 63-nucleotide fragment within the UTR and achieves a sensitivity of up to 10 viral genomes per mL of plasma or whole blood. The potential for inhibition was evaluated during the testing process. Inhibition was assessed by spectrophotometric analysis of extracted DNA (A260/280 ratio) and by spiking TTV-negative samples with plasmid DNA containing the target sequence (100 copies/μL, ~10× the detection limit) prior to extraction. Inhibition rates were calculated based on failure to detect the target DNA-containing plasmid. The lower limit of detection was 1.0 log DNA copies/mL. TTV viremia equal to or exceeding 10,000 copies/mL (≥4 log copies/mL) was categorized as high-level viremia.

HSV-1 and VZV viremia were assessed in a subsample of 456 patients (241 females and 215 males; mean age, 82.6 ± 7.3 years) using quantitative real-time PCR assays. Viral DNA was analyzed using the GeneProof Herpes Simplex Virus 1 PCR Kit and the GeneProof Varicella-Zoster Virus PCR Kit (GeneProof a.s., Brno, Czech Republic), according to the manufacturer’s instructions. The HSV-1 assay targets the glycoprotein B (gB) gene, whereas the VZV assay targets a conserved sequence within the single-copy ORF62 gene. Both assays are based on TaqMan hydrolysis probe technology and include an Internal Standard to monitor DNA extraction efficiency and PCR inhibition. Viral DNA loads were quantified using the calibration standards supplied with each kit and expressed as copies/mL. The lower limit of detection was approximately 272.6 copies/mL for HSV-1 and 119.9 copies/mL for VZV, with quantitative linear ranges extending up to 10^10^ copies/mL. The characteristics of participants with available HSV-1/VZV DNAemia measurements were compared with those of the remaining cohort to assess the comparability of the subsample with the overall study population (Supplementary Table S1).

### 2.3. Blood Bacterial DNA (BB-DNA) Quantification

DNA was isolated from 300 µL of whole blood using the QIAamp DNA Blood Mini Kit (Qiagen GmbH, Hilden, Germany) following the manufacturer’s protocol. Blood bacterial DNA (BB-DNA) levels were determined by quantitative real-time PCR amplification of the bacterial 16S rRNA gene, as previously described [12]. In brief, real-time qPCR assays were performed using universal primers targeting the V3–V4 hypervariable region of the 16S rRNA gene. Each 20 µL reaction mixture contained 20 ng of template DNA, 1× SensiFAST SYBR Hi-ROX Mix (Bioline, London, UK), and 0.4 µM of each primer (forward: 5′-TCCTACGGGAGGCAGCAGT-3′; reverse: 5′-GGACTACCAGGGTATCTAATCCTGTT-3′). PCR amplification was carried out with an initial enzyme activation step at 95 °C for 2 min, followed by 40 cycles consisting of denaturation at 95 °C for 15 s and annealing/extension at 60 °C for 60 s. A melting curve analysis (60–95 °C) was subsequently performed to verify amplicon specificity. Quantification was based on fluorescence measurements acquired during the exponential phase of amplification. For absolute quantification, standard curves were generated in each run using 10-fold serial dilutions of bacterial DNA reference standards (Femto Bacterial DNA Quantification Kit, Zymo Research), covering concentrations from 0.0002 to 2 pg, and were compared with previously established curves to ensure inter-assay reproducibility. All reactions were run in triplicate using a QuantStudio 3 Real-Time PCR System (Thermo Fisher Scientific, Segrate, Milan, Italy). BB-DNA concentrations were expressed as ng/mL of whole blood after normalization for the DNA extraction dilution factor and the initial volume of blood processed.

### 2.4. Automated Immunoassay for Serum Biomarker Assessment

Serum concentrations of interleukin-6 (IL-6), interleukin-10 (IL-10), interleukin-17A (IL-17A), growth differentiation factor 15 (GDF15), neurofilament light chain (NfL), osteopontin (OPN), C-C motif chemokine ligand 22 (CCL22), fibroblast growth factor 21 (FGF21), follistatin (FST), were determined in triplicate using ELLA™ microfluidic immunoassay platform (ProteinSimple, Bio-Techne, San Jose, CA, USA), following the manufacturer’s recommended procedure. The analytical sensitivities of the assays were as follows: IL-6, 0.11 pg/mL; IL-10, 0.17 pg/mL; IL-17A, 0.10 pg/mL; GDF15, 0.21 pg/mL; NfL, 1.09 pg/mL; OPN, 7.33 pg/mL; CCL22, 0.32 pg/mL; FGF21, 3.74 pg/mL; FST, 1.09 pg/mL. For all biomarkers, intra-assay coefficients of variation (CVs) were <10%. Calibration curves were generated for each cartridge by the manufacturer and used according to the standard operating procedure.

### 2.5. Telomere Length Assessment

Leukocyte telomere length (LTL) was measured in a subsample of 872 participants representative of the overall cohort in terms of age distribution, sex, and clinical characteristics for whom LTL measurements were available, as previously described [41]. LTL was determined by quantitative real-time PCR (qPCR) and expressed as the telomere-to-single-copy gene (T/S) ratio. The assay was performed according to the method described by Cawthon [42] with minor adaptations. The acidic ribosomal phosphoprotein P0 gene (36B4) was used as the single-copy reference gene. Primer sequences and concentrations were as previously reported [42]. Each qPCR reaction contained 20 ng of genomic DNA in a final reaction volume of 15 µL (5 µL DNA and 10 µL master mix). A five-point standard curve was generated using serial 1.68-fold dilutions of a reference DNA sample, covering an input range of 2–30 ng per reaction. To limit inter-run variability, telomere and 36B4 amplifications were performed on the same plate whenever possible, using the same calibrator DNA sample (Roche, Milan, Italy) across runs. The qPCR protocol began with a 10-s denaturation step at 95 °C, followed by 30 amplification cycles consisting of denaturation at 95 °C for 5 s, annealing at 57 °C for 15 s, and extension at 72 °C for 20 s. Reactions were run on a Rotor-Gene real-time PCR system (Qiagen). Each sample was analyzed in duplicate, and replicate measurements were considered acceptable when the difference in cycle threshold (Ct) values was less than 0.5 cycles. Samples exceeding this threshold were reanalyzed. Relative telomere length was expressed as the normalized T/S ratio.

### 2.6. Statistical Analysis

Continuous variables were summarized using mean ± standard error of the mean (SEM), whereas categorical variables were presented as percentages. The distribution of continuous variables was assessed with the one-sample Kolmogorov–Smirnov test. Variables showing a non-normal distribution were subsequently subjected to logarithmic transformation before parametric analyses. Differences among groups were evaluated using analysis of covariance (ANCOVA) for continuous variables adjusted for age and sex, and Pearson’s χ^2^ test for categorical variables (Table 1). For continuous biological measures, differences according to immunonutritional status (PNI < 40 vs. PNI ≥ 40) and across tertiles of circulating bacterial DNA (BB-DNA) were examined using analysis of covariance (ANCOVA). When appropriate, significant overall differences were further explored through pairwise post-hoc comparisons. The assumptions of homogeneity of variances and homogeneity of regression slopes were assessed for the ANCOVA models. Homogeneity of variances was evaluated using Levene’s test, while homogeneity of regression slopes was assessed by testing the interaction between the continuous covariates included in the models and the corresponding grouping factor. No meaningful violations of these ANCOVA assumptions were identified. ANCOVA models were adjusted for age, sex, frailty status, Charlson Comorbidity Index (CCI), infections and creatinine levels. These covariates were selected a priori based on their established or clinically plausible relationships with immunonutritional status, systemic inflammation, and clinical vulnerability in hospitalized older adults. The strength and direction of monotonic relationships between continuous parameters (e.g., BB-DNA levels and TTV viremia) were determined using the Spearman’s rank correlation coefficient.

**Figure 1:**
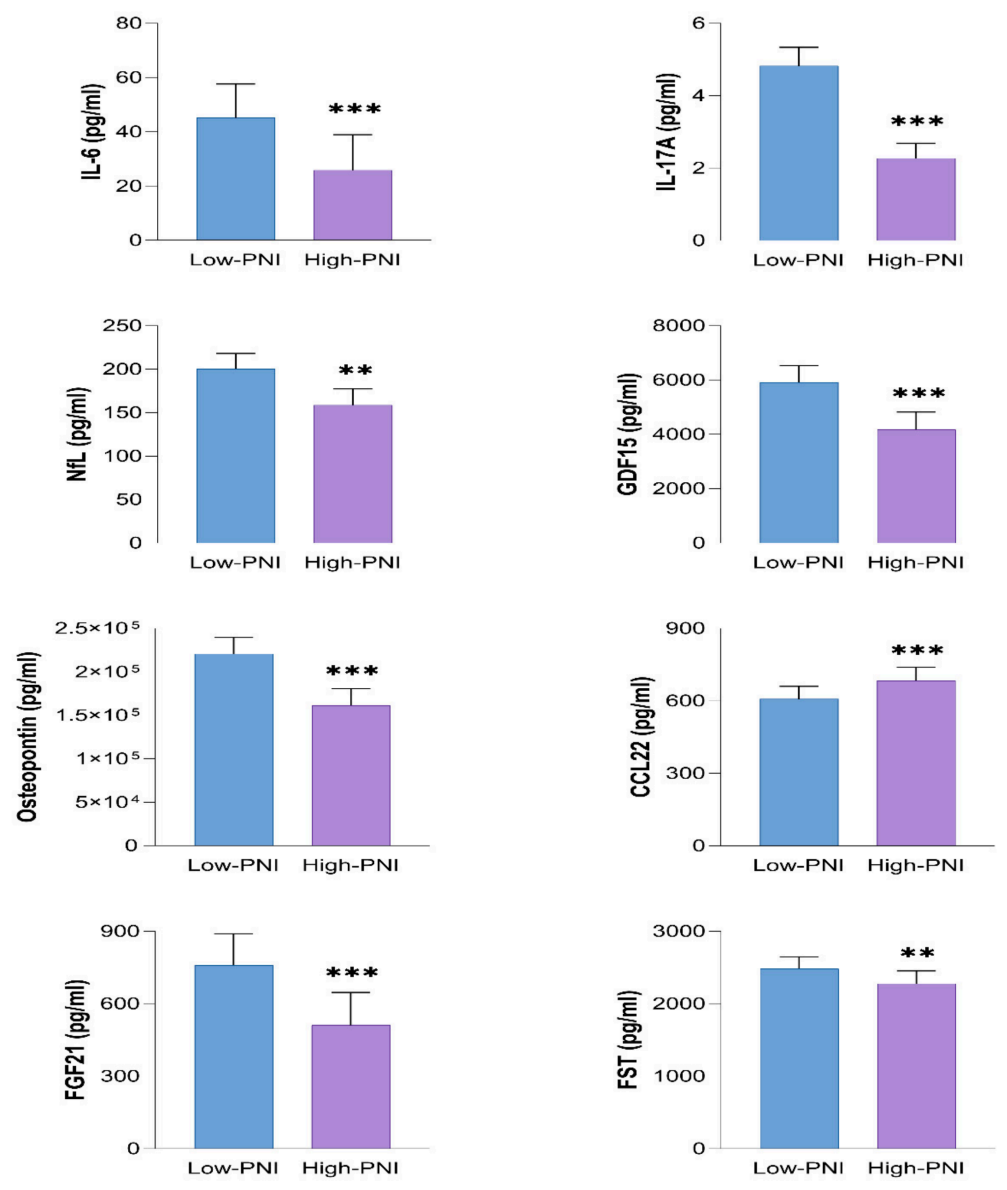
Circulating levels of inflammatory, metabolic, and neurodegenerative biomarkers according to immunonutritional status. Comparison of serum interleukin-6 (IL-6), interleukin-17A (IL-17A), neurofilament light chain (NfL), growth differentiation factor 15 (GDF15), osteopontin, C-C motif chemokine ligand 22 (CCL22), fibroblast growth factor 21 (FGF21), and follistatin (FST) between hospitalized older adults stratified into the low-PNI group (PNI < 40) and high-PNI group (PNI ≥ 40). Statistical significance was evaluated by ANCOVA adjusted for age, sex, frailty status, CCI, infections and creatinine levels (** *p* < 0.01, *** *p* < 0.001, FDR *p*.adj < 0.01).

**Figure 2:**
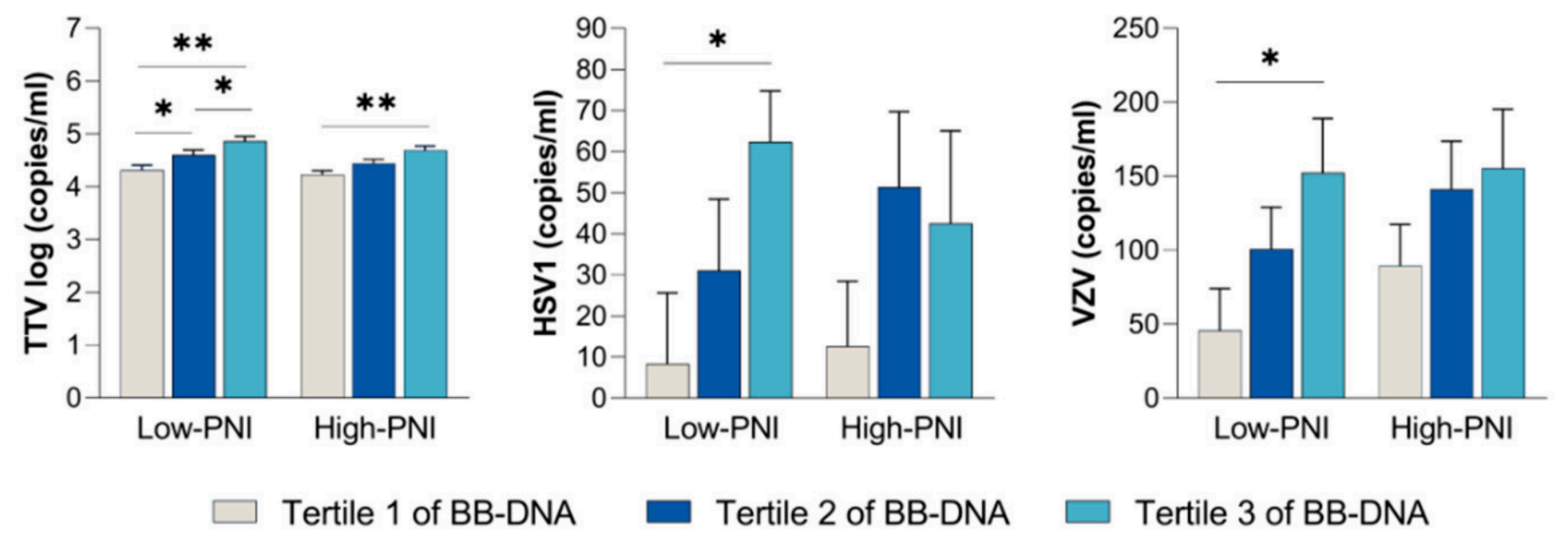
Viral DNA loads across circulating bacterial DNA (BB-DNA) tertiles stratified by immunonutritional status. Quantification of Torque teno virus (TTV), Herpes simplex virus 1 (HSV-1), and Varicella-zoster virus (VZV) DNA copies across low (T1), intermediate (T2), and high (T3) tertiles of BB-DNA in low-PNI and high-PNI patients. Brackets indicate statistically significant differences between tertiles (* *p* < 0.05, ** *p* < 0.01, FDR *p*.adj < 0.05).

**Figure 3:**
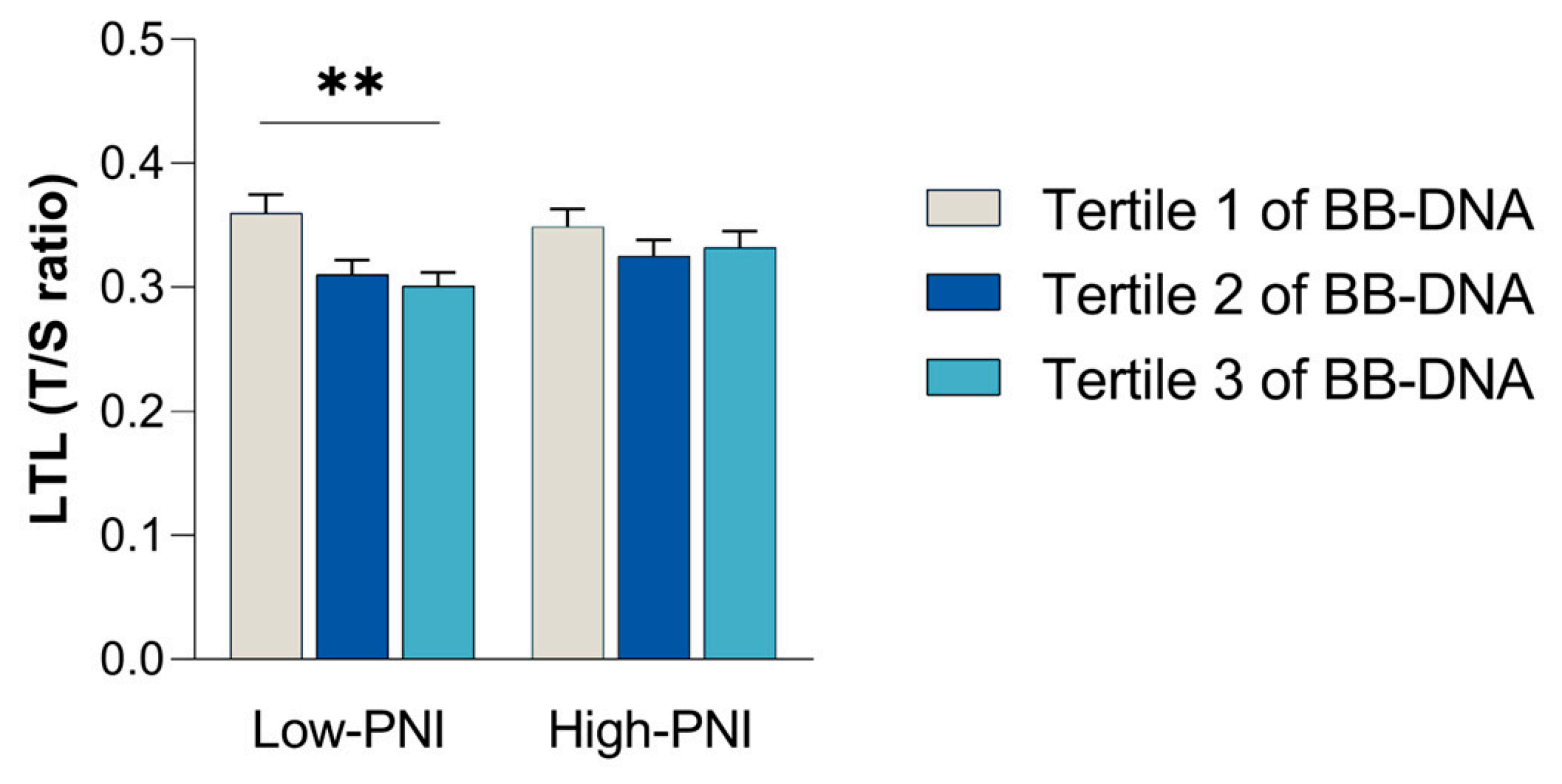
Leukocyte telomere length across circulating bacterial DNA (BB-DNA) tertiles by immunonutritional status. Leukocyte telomere length (LTL) was compared across low (T1), intermediate (T2), and high (T3) BB-DNA tertiles according to Prognostic Nutritional Index (PNI) category (low vs. high). Brackets indicate statistically significant differences between tertiles within the low-PNI group (** *p* < 0.01). These exploratory analyses were not subjected to multiplicity adjustment.

**Figure 4:**
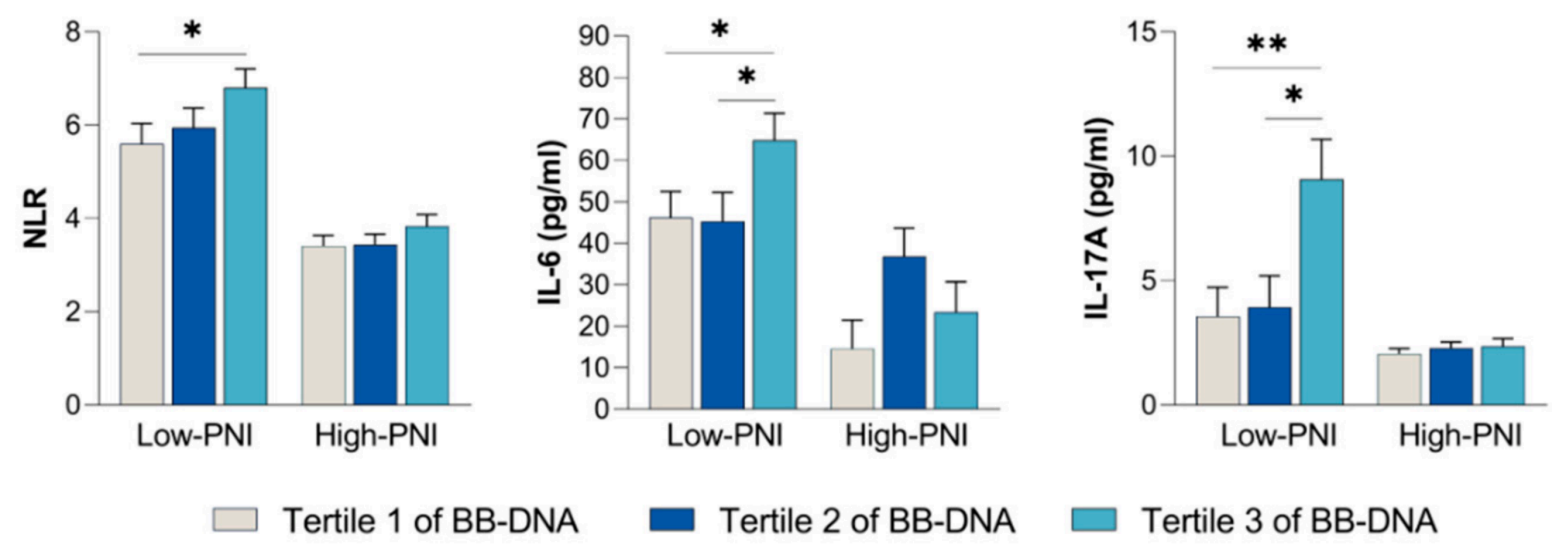
Host inflammatory markers across circulating bacterial DNA (BB-DNA) tertiles stratified by immunonutritional status. Levels of the neutrophil-to-lymphocyte ratio (NLR), interleukin-6 (IL-6), and interleukin-17A (IL-17A) were compared across low (T1), intermediate (T2), and high (T3) BB-DNA tertiles according to Prognostic Nutritional Index (PNI) category (low vs. high). Brackets indicate statistically significant differences between tertiles (* *p* < 0.05, ** *p* < 0.01). These exploratory analyses were not subjected to multiplicity adjustment.

Multivariable logistic regression analyses were performed to evaluate the independent associations of log-transformed BB-DNA levels or BB-DNA tertiles and low-PNI status with cognitive impairment and individual interRAI-derived neurocognitive and behavioral conditions. Covariates were selected a priori to account for major demographic, clinical vulnerability, comorbidity, renal function, medication, and inflammatory/hematological factors that could plausibly confound these associations. Odds ratios (ORs) and corresponding 95% confidence intervals (CIs) were calculated using four models: Model 1 was adjusted for age, sex, number of medications at admission, and estimated glomerular filtration rate (eGFR); Model 2 was additionally adjusted for the neutrophil count; and Model 3 was further adjusted for the CCI; Model 4 additionally included the Frailty Index as a further sensitivity analysis. These additional adjustments were performed because of the potential overlap between comorbidity burden, frailty, and cognitive outcomes. The progressive modelling strategy was used to assess the robustness of the associations after accounting for demographic, renal, inflammatory, and comorbidity-related factors. Multicollinearity among covariates was assessed using the variance inflation factor (VIF). Effect modification by PNI status was formally assessed by including BB-DNA × PNI interaction terms in the corresponding models. Information on standardized acute illness severity scores, specific admission diagnoses, antibiotic exposure, and immunosuppressive therapy was not systematically available in the dataset used for these analyses. These factors were therefore not included as covariates and may re-present sources of residual confounding. Similarly, liver disease was not available as a standardized covariate for the present analyses. To balance the control of Type I error with the exploratory nature of the multiple biological and clinical assessments, a dual statistical framework was applied:

Primary analyses: The primary analyses evaluated the associations of PNI-defined immunonutritional status with the two main outcome domains of the study: circulating bacterial DNA (BB-DNA) and neurocognitive vulnerability. For the primary comparisons of biomarker profiles between PNI groups (Figure 1), and analyses of viral DNAemia across BB-DNA tertiles within PNI strata (Figure 2) *p*-values were adjusted for multiple testing using the Benjamini–Hochberg false discovery rate (FDR) procedure. An FDR-adjusted *p*-value < 0.05 was considered statistically significant. Statistical analyses of neurocognitive outcomes were conducted using the models described above. Additional exploratory analyses: Analyses of inflammatory markers (IL-6, IL-17A, and NLR) and the cellular aging marker LTL across BB-DNA tertiles within PNI strata (Figure 3 and Figure 4), as well as analyses of domain-specific neurocognitive items (Table A2), were considered exploratory and hypothesis-generating. These analyses were not subjected to additional multiplicity adjustment, and nominal (unadjusted) *p*-values < 0.05 were considered statistically significant. Interpretation of exploratory findings was based primarily on the magnitude and direction of the observed effects, together with their corresponding 95% confidence intervals, rather than on statistical significance alone. All analyses were performed using IBM SPSS Statistics for Windows, version 29.0.1.0 (171) (IBM Corp., Armonk, NY, USA).

## 3. Results

### 3.1. Clinical Characteristics of the Study Population by PNI Group

A total of 1016 hospitalized older adults from the Report-AGE cohort were included in the analysis. According to the PNI classification, 494 patients were assigned to the low-PNI group (Group 1) and 522 to the high-PNI group (Group 2). The baseline clinical and laboratory characteristics of the study population are summarized in Table 1.

Patients in the low-PNI group were significantly older than those in the high-PNI group (85.9 ± 5.6 vs. 80.8 ± 7.4 years, *p* < 0.001) and had a higher burden of age-related comorbidities. Atrial fibrillation (24.7% vs. 14.6%), chronic kidney disease (34.0% vs. 11.9%), chronic obstructive pulmonary disease (19.0% vs. 11.3%), and infections (31.8% vs. 9.2%) were all more prevalent in the low-PNI group (*p* < 0.001). No significant differences were observed for diabetes, hypertension, ischemic heart disease, cancer, or dementia.

Immunonutritional impairment was also associated with a higher frailty burden. Frailty distribution differed markedly between groups, with a significantly higher proportion of frail individuals (FI ≥ 0.20) in the low-PNI group (52.0% vs. 35.4%, *p* < 0.001) and a lower prevalence of non-frail individuals (15.4% vs. 23.8%, *p* < 0.001).

Laboratory analyses revealed that patients with lower PNI values exhibited a profile consistent with enhanced systemic inflammation and impaired hematological status (Table 1). After adjustment for age and sex, patients with low-PNI showed lower red blood cell counts (*p* < 0.001), reduced hemoglobin concentrations (*p* < 0.001), and lower lymphocyte counts (*p* < 0.001), together with increased neutrophil (*p* < 0.001) and monocyte counts (*p* < 0.05). Consistently, albumin levels were lower in the low-PNI group (*p* < 0.001).

Markers of inflammation and organ dysfunction were elevated in the low-PNI group. C-reactive protein concentrations were approximately five-fold higher (*p* < 0.001), while renal function was significantly worse, as indicated by higher creatinine levels and lower eGFR (*p* < 0.001 and *p* < 0.01 respectively).

Mean BB-DNA concentrations were significantly higher in patients with lower PNI values than in those with better immunonutritional status (53.7 ± 3.5 vs. 42.2 ± 2.9 ng/mL, *p* < 0.05).

### 3.2. Poor Immunonutritional Status Is Associated with Biomarkers of Immune Activation, Systemic Stress, and Tissue Damage

Immunonutritional impairment was associated with an unfavorable biomarker profile related to systemic stress, immune dysregulation, and neurodegeneration (Figure 1). Patients with lower PNI values exhibited significantly higher circulating levels of growth differentiation factor 15 (GDF15) (*p* < 0.001), osteopontin (OPN) (*p* < 0.001), fibroblast growth factor 21 (FGF21) (*p* < 0.001), follistatin (FST) (*p* < 0.01), and neurofilament light chain (NfL) (*p* < 0.01). In parallel, lower PNI values were associated with higher levels of immune and inflammatory mediators, including interleukin-6 (IL-6), and interleukin-17A (IL-17A) and reduced C-C motif chemokine ligand 22 (CCL22), (all *p* < 0.001) (Figure 1).

### 3.3. Viral Loads and Inflammatory Markers Across BB-DNA Tertiles According to Immunonutritional Status

To investigate the relationship between BB-DNA levels, viral DNAemia, and inflammatory and cellular aging markers, patients were stratified into BB-DNA tertiles within each PNI group, defined as low (T1), intermediate (T2), and high (T3) BB-DNA levels (Figure 2, Figure 3 and Figure 4). HSV-1 and VZV DNAemia analyses were restricted to the 456 participants with available measurements. These participants were broadly comparable with the remaining 560 participants with respect to age, sex, PNI, frailty, and major comorbidities, although a lower prevalence of infections was observed in the HSV-1/VZV subsample (16.0% vs. 23.6%, *p* < 0.01) (Supplementary Table S1).

In the low-PNI group, TTV viremia increased progressively across BB-DNA tertiles (T1 vs. T2, T2 vs. T3, *p* < 0.05 and T1 vs. T3, *p* < 0.01), with the T1–T3 comparison remaining significant after FDR adjustment (FDR < 0.05). In the high-PNI group, TTV viremia differed significantly only between T1 and T3 (*p* < 0.01, FDR < 0.05) (Figure 2).

**Figure A2:**
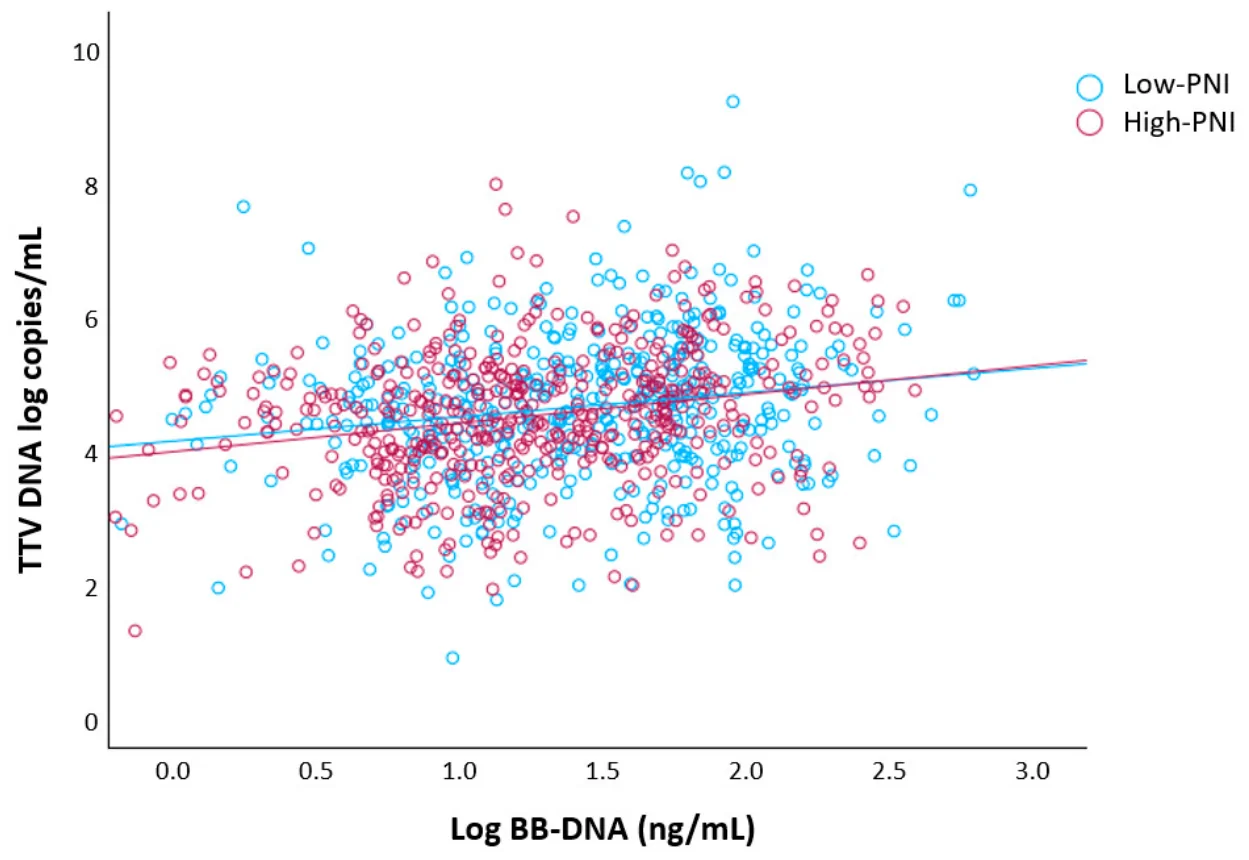
Correlation between log-transformed TTV viral load and log-transformed BB-DNA levels according to immunonutritional status. BB-DNA levels were positively correlated with TTV viremia in both low-PNI (ρ = 0.197, *p* < 0.001) and high-PNI patients (ρ = 0.247, *p* < 0.001). Data are stratified by immunonutritional status. The blue line represents the low-PNI group, and the red line represents the high-PNI group.

A similar pattern was observed for herpesvirus DNAemia. In the low-PNI group, both HSV-1 and VZV DNAemia increased progressively across BB-DNA tertiles, with significantly higher levels in T3 compared with T1 (*p* < 0.05, FDR-adjusted *p* < 0.05, for both comparisons), whereas these differences were attenuated or absent in the high-PNI group (Figure 2). Formal interaction analyses did not support a statistically significant difference in these associations according to PNI status. Consistent with these findings, BB-DNA levels were positively correlated with TTV viremia in both low-PNI (rho = 0.197, *p* < 0.001) and high-PNI patients (rho = 0.247, *p* < 0.001) (Figure A2). TTV viremia tended to be higher in the low-PNI group than in the high-PNI group (4.610 ± 0.055 vs. 4.474 ± 0.047 log copies/mL; *p* = 0.066), after adjustment for age, sex, frailty status, CCI, infections, and creatinine levels. Given the exploratory nature of the analyses shown in Figure 3 and Figure 4, nominal *p*-values are reported for these comparisons. These findings were interpreted primarily according to the magnitude and direction of the observed differences and their biological consistency. Increasing BB-DNA was associated with differences in LTL and inflammatory markers in the stratified analyses, with several nominally significant associations observed in the low-PNI group. In stratified analyses, LTL progressively decreased across increasing BB-DNA tertiles within the low-PNI group (*p* < 0.01), whereas no significant differences across BB-DNA tertiles were observed within the high-PNI group (Figure 3). LTL values were comparable between the low- and high-PNI groups (0.324 ± 0.008 vs. 0.327 ± 0.008). These stratified findings describe an association between BB-DNA and LTL within the low-PNI group but do not demonstrate that the BB-DNA–LTL association differs according to PNI status (BB-DNA × PNI interaction: *p* = 0.557). Among inflammatory and immunosenescence-related markers, nominally significant differences across BB-DNA tertiles were observed in the low-PNI group for NLR (T1 vs. T3, *p* < 0.05), IL-6 (T1 vs. T3 and T2 vs. T3, *p* < 0.05), and IL-17A (T1 vs. T3, *p* < 0.01; T2 vs. T3, *p* < 0.05) (Figure 4). No clear differences across BB-DNA tertiles were observed for the other mediators (GDF15, OPN, CCL22, NfL, FGF21, and FST; Table A1). However, formal BB-DNA×PNI interaction analyses did not identify significant interactions for TTV, HSV-1, or VZV DNAemia, indicating that the observed differences in the stratified analyses should not be interpreted as evidence of effect modification by PNI.

### 3.4. Poor Immunonutritional Status and BB-DNA Show Distinct Associations with Neurocognitive and Behavioral Conditions

In multivariable logistic regression analyses (Table 2), BB-DNA and poor immunonutritional status exhibited distinct patterns of association with neurocognitive and behavioral conditions. An independent association was observed between higher BB-DNA levels and acute change in mental status from baseline (ACMS) across all models (Model 1: OR 1.93, 95% CI 1.14–3.29; Model 2: OR 1.75, 95% CI 1.09–2.81; Model 3: OR 1.74, 95% CI 1.08–2.79; Model 4: OR 1.76, 95% CI 1.09–2.82), whereas low-PNI was not associated with ACMS. Both BB-DNA and low-PNI were independently associated with behavioral disturbances (Model 2: OR 3.09, 95% CI 1.11–8.59 and OR 1.80, 95% CI 1.05–3.06, respectively). BB-DNA levels were not associated with cognitive impairment (Model 2: OR 0.98, 95% CI 0.77–1.22). Conversely, low-PNI was independently associated with an increased risk of cognitive impairment in the primary model (Model 2: OR 1.39, 95% CI 1.06–1.83). This association remained essentially unchanged after additional adjustment for CCI and the Frailty Index (Model 3: OR 1.39, 95% CI 1.05–1.82; Model 4: OR 1.42, 95% CI 1.08–1.88). Low-PNI was also independently associated with easily distracted behavior (Model 2: OR 1.75, 95% CI 1.29–2.35), whereas no significant associations were observed between either BB-DNA or PNI and episodes of disorganised speech (EDS) or mental function varying over the course of the day (MFV).

To assess the robustness of these findings, we performed a sensitivity analysis with additional adjustment for infection, together with medication count, eGFR, neutrophil count, CCI, and frailty (Supplementary Table S2). The associations of BB-DNA with ACMS (OR 1.70, 95% CI 1.04–2.75) and behavioral disturbances (OR 3.16, 95% CI 1.07–9.04) remained statistically significant. Similarly, low-PNI remained associated with easily distracted behavior (OR 2.04, 95% CI 1.47–2.84), behavioral disturbances (OR 1.87, 95% CI 1.07–3.28), and cognitive impairment (OR 1.49, 95% CI 1.08–2.05). No significant associations were observed between BB-DNA and cognitive impairment or easily distracted behavior, or between either predictor and EDS or MFV.

To gain further insight into the specific cognitive domains associated with immunonutritional status, we conducted additional exploratory multivariable logistic regression analyses focusing on memory sub-domains at hospital admission (Table A2). These analyses included the entire study cohort (*N* = 1016). Low-PNI was associated with short-term memory impairment across the multivariable models, including the fully adjusted model (Model 4: OR 1,90, 95% CI 1.42–2.57). Regarding situational memory, low PNI was significantly associated with the outcome in both Model 1 (OR 1.46, 95% CI 1.01–2.11) and Model 2 (OR 1.44, 95% CI 1.02–2.15). However, the association was attenuated and no longer statistically significant after additional adjustment for CCI and the Frailty Index (Model 3: OR 1.43, 95% CI 0.98–2.11; Model 4: OR 1.49, 95% CI 0.99–2.23). No significant associations were observed for procedural memory across any of the models. Furthermore, circulating BB-DNA did not show any significant association with any of the three specific memory domains evaluated at admission (Table A2).

## 4. Discussion

Among hospitalized older adults included in this study, poor immunonutritional status, measured by the Prognostic Nutritional Index (PNI), was associated with a broad clinical and biological vulnerability profile, including frailty, multimorbidity, systemic inflammation, increased circulating bacterial DNA (BB-DNA), cognitive impairment, and higher levels of biomarkers related to tissue damage and neurodegeneration. Overall, our findings suggest that immunonutritional impairment is associated with a clinical and biological profile encompassing higher circulating bacterial DNA levels, inflammatory alterations, and neurocognitive vulnerability. Associations involving viral DNAemia were additionally observed in analyses stratified by PNI. However, these results do not establish a common underlying mechanism or temporal sequence. Patients with low-PNI exhibited higher BB-DNA levels, elevated inflammatory markers, and a greater burden of frailty and comorbidity. These observations are consistent with the concept that malnutrition may contribute to immune dysregulation and impaired gut barrier integrity [15,43,44]. Aging is characterized by changes in epithelial barrier function and mucosal immune surveillance, including reduced tight-junction protein expression and impaired intestinal immune responses [45,46]. Nutritional deficiencies may further exacerbate these alterations, potentially contributing to increased passage of microbial products into the circulation and systemic inflammation, as previously described in older individuals with dementia [47]. Experimental evidence from vitamin D3-deficient models similarly supports a link between impaired barrier function, altered host–microbial homeostasis, and reduced antiviral defenses [48]. However, the direction of this relationship cannot be established in the present cross-sectional study. In hospitalized older adults, acute illness, infection, systemic inflammation, organ dysfunction, and other clinical stressors may contribute to reductions in serum albumin and lymphocyte counts and consequently to lower PNI, while impaired nutritional and immunological status may also be associated with inflammatory and barrier-related alterations. Thus, reverse causality and bi-directional relationships cannot be excluded.

A particularly novel aspect of our findings is the relationship between immunonutritional status, BB-DNA, and viral DNAemia. Among participants with low-PNI, increasing BB-DNA levels were associated with higher TTV viral load, as well as with progressively higher HSV-1 and VZV DNAemia, whereas these patterns were attenuated or absent in patients with better immunonutritional status. These associations were observed in the primary analyses of viral DNAemia, with the relevant comparisons remaining significant after FDR correction, supporting the robustness of the observed pattern across the biomarker comparisons. The positive association between BB-DNA and TTV is particularly relevant, as TTV has emerged as a potential marker of immune impairment and has been associated with mortality in older adults [21,23,49]. Previous studies have also linked herpesvirus reactivation to poor clinical outcomes in critically ill patients [50,51]. However, in our patient setting, HSV-1 and VZV DNAemia should be interpreted as evidence of detectable circulating viral DNA rather than as direct evidence of a longitudinal viral reactivation process, since viral DNA was assessed at a single time point. Together, these findings are consistent with a possible relationship between bacterial and viral DNAemia in hospitalized older adults with impaired immunonutritional status, but do not establish whether these processes are temporally or biologically linked. Accelerated cellular aging may represent an additional component of this process. In stratified analyses, LTL progressively decreased across increasing BB-DNA tertiles in the low-PNI group, consistent with previous reports linking microbial translocation to telomere shortening [52]. Chronic inflammatory activation has also been associated with reduced telomerase reverse transcriptase (hTERT) expression and accelerated telomere attrition, supporting a link between microbial-inflammatory burden and cellular aging [53].

Because the analyses of LTL and inflammatory markers across BB-DNA tertiles were exploratory and based on nominal *p*-values, these findings should be considered hypothesis-generating. Nevertheless, the direction and magnitude of the observed differences are biologically consistent with a possible relationship between systemic inflammatory burden and cellular aging. Beyond microbial and viral markers, low-PNI was associated with a broad molecular signature of systemic stress.

Patients with lower PNI values exhibited higher circulating concentrations of IL-6, IL-17A, GDF15, OPN, FGF21, FST, and NfL, together with reduced CCL22 levels, indicating a biomarker profile consistent with inflammatory activation, metabolic stress, impaired immune regulation, and neuroaxonal damage. In particular, elevated GDF15 and FGF21 may reflect ongoing catabolic remodeling and mitochondrial stress, whereas increased NfL may indicate axonal injury; these processes have been previously linked to frailty, cognitive impairment, and adverse aging trajectories [54,55,56,57,58,59,60]. Reduced CCL22 levels in low-PNI patients were observed despite increased circulating monocytes. This pattern may indicate impaired chemokine-mediated immune regulation, consistent with experimental evidence showing altered macrophage function during protein malnutrition in aged animals [61,62]. These biomarker differences should nevertheless be interpreted in the context of the multifactorial clinical condition of hospitalized older adults, in whom inflammation, comorbidity, organ dysfunction, and nutritional status are closely interconnected.

Low-PNI was independently associated with cognitive impairment, easily distracted behavior and behavioral disturbances, after adjustment for age, sex, frailty, comorbidity burden, renal function, and inflammatory status. Additional exploratory analyses of specific cognitive domains identified an association between low-PNI and impaired short-term memory that persisted after multivariable adjustment, whereas the association with situational memory was attenuated and no longer statistically significant after additional adjustment for comorbidity and frailty. Procedural memory showed no association with PNI. The association between low-PNI and easily distracted behavior is of particular interest, as it suggests that impaired immunonutritional status may also be related to attentional processes even in the absence of acute confusional changes. These observations are consistent with previous studies linking malnutrition to cognitive impairment [63,64,65,66] and suggest that immunonutritional impairment may be associated with global cognitive vulnerability, while the domain-specific memory findings should be considered exploratory and interpreted with caution.

In contrast, BB-DNA was independently associated with ACMS and behavioral disturbances, but not with baseline cognitive impairment or the other interRAI neurocognitive items, suggesting that circulating bacterial DNA may be more closely linked to acute brain dysfunction than to the baseline cognitive impairment assessed at hospital admission. Acute confusional status and behavioral disturbances are considered manifestations of brain vulnerability triggered by systemic insults, particularly inflammatory stimuli, consistent with experimental evidence demonstrating that IL-1β-driven neuroinflammation can induce behavioral and cognitive dysfunction [67,68]. Bacterial DNA and microbial products can activate innate immune pathways, leading to cytokine release, endothelial dysfunction, and blood–brain barrier disruption, all of which are implicated in delirium pathophysiology [69]. Our findings are therefore compatible with a relationship between higher BB-DNA levels and acute neurocognitive dysfunction during hospitalization, but they do not establish BB-DNA as a mediator or cause of these manifestations. These results differ from those previously reported by our group among community-dwelling older individuals with mild cognitive impairment or Alzheimer’s disease, where BB-DNA was associated with longitudinal cognitive decline, possibly reflecting differences in disease stage, clinical setting, outcome definition, and study design [70]. Taken together, these findings suggest that poor immunonutritional status and higher BB-DNA may identify partially distinct dimensions of neurocognitive vulnerability during hospitalization. While low-PNI was associated with global and selected domain-specific cognitive vulnerability, BB-DNA was associated with acute confusional and behavioral manifestations, supporting the hypothesis that circulating microbial products may be relevant to acute brain dysfunction in hospitalized older adults. This study has several limitations. First, the predominantly cross-sectional nature of this observational study prevents establishing causal relationships and precludes determining the temporal or directional sequence linking low-PNI, microbial translocation, viral DNAemia, systemic inflammation, and neurocognitive alterations. In particular, acute illness severity and systemic inflammation may simultaneously influence serum albumin, lymphocyte counts, circulating bacterial DNA, viral DNAemia, and cognitive status, resulting in associations that cannot be interpreted as evidence of a specific biological sequence. Second, circulating bacterial 16S rRNA gene DNA (BB-DNA) was used as an indirect marker of circulating bacterial burden and does not by itself demonstrate increased intestinal permeability, microbial translocation, or a gastrointestinal source of the detected bacterial DNA. Direct measures of intestinal permeability and endotoxemia were not available in the present study. Third, no information on gut microbiome composition was collected, preventing identification of specific microbial taxa or community signatures associated with the observed findings. Fourth, viral DNAemia was assessed at a single time point and may not capture temporal fluctuations in viral burden or distinguish transient DNAemia from sustained viral reactivation. Fifth, because serum albumin concentrations and lymphocyte counts are influenced by factors beyond nutritional status, the PNI cannot be considered a specific or exclusive marker of nutrition. Although originally developed to assess nutritional status, PNI values are substantially affected by a range of clinical conditions, including malignancy, acute or chronic infections, immunosuppression, and impaired liver or kidney function. Sixth, patients who died during hospitalization were excluded because the predefined study assessments and/or required measurements were unavailable or incomplete in these individuals. Although this exclusion was based on the availability of standardized assessments, it may have introduced some survivor or selection bias, since in-hospital mortality may be related to nutritional status, inflammation, infection, and systemic biomarkers. Finally, residual confounding related to acute illness severity and unmeasured clinical factors cannot be excluded. In particular, standardized measures of acute illness severity, admission diagnosis, antibiotic exposure, and immunosuppressive treatment were not systematically available and could not therefore be incorporated into the multivariable models. The generalizability of these findings may be limited because the study population consisted of older adults admitted to hospital; therefore, the results may not be directly applicable to older adults living in the community. Longitudinal studies incorporating repeated measurements of PNI, microbial and viral markers, inflammatory mediators, and neurocognitive outcomes are needed to clarify temporal relationships and assess whether the observed associations persist beyond acute illness.

## 5. Conclusions

Low-PNI in hospitalized older adults was associated with increased circulating bacterial DNA, systemic inflammatory alterations, and neurocognitive vulnerability, while higher BB-DNA levels were associated with viral DNAemia, selected inflammatory markers, shorter leukocyte telomere length, and acute neurocognitive and behavioral manifestations. Although the cross-sectional design does not establish temporal or causal relationships, these findings suggest that poor immunonutritional status, bacterial and viral burden, systemic inflammation, and neurocognitive alterations may represent interconnected features of clinical vulnerability in hospitalized older adults. Longitudinal studies are needed to clarify the temporal relationships among these factors and to determine their clinical and biological significance.

## Figures and Tables

**Table 1:** Characteristics of older hospitalized patients.

	Group 1 (Low-PNI) *N* = 494	Group 2 (High-PNI) *N* = 522	*p* Value
Age (yrs), mean ± SD	85.9 ± 5.6	80.8 ± 7.4	<0.001
Males, *n* (%)	217 (43.9%)	255 (48.9%)	0.116
Diabetes, *n* (%)	108 (21.9%)	115 (22.0%)	0.948
Hypertension, *n* (%)	308 (62.3%)	312 (59.7%)	0.400
AF, *n* (%)	122 (24.7%)	76 (14.6%)	<0.001
IHD, *n* (%)	73 (14.8%)	64 (12.3%)	0.240
CKD, *n* (%)	168 (34.0%)	62 (11.9%)	<0.001
COPD, *n* (%)	94 (19.0%)	59 (11.3%)	<0.001
Cancer, *n* (%)	71 (14.4%)	81 (15.5%)	0.609
Infections, *n* (%)	157 (31.8%)	48 (9.2%)	<0.001
Dementia, *n* (%)	80 (16.2%)	104 (19.9%)	0.123
Frailty Index, *n* (%)			<0.001
<0.10	76 (15.4%)	124 (23.8%)	
0.10–0.19	161 (32.6%)	213 (40.8%)	
≥0.20	257 (52.0%)	185 (35.4%)	
RBC (×10^6^/µlL)°, mean ± SEM	3.76 ± 0.07	4.28 ± 0.07	<0.001
Hemoglobin (g/dl)°, mean ± SEM	10.9 ± 0.2	12.8 ± 0.2	<0.001
Neutrophils (×10^3^/μL)°, mean ± SEM	6.76 ± 0.42	5.35 ± 0.44	<0.001
Lymphocytes (×10^3^/μL)°, mean ± SEM	1.42 ± 0.10	1.63 ± 0.10	<0.001
Monocytes (×10^3^/μL)°, mean ± SEM	0.60 ± 0.02	0.54 ± 0.02	0.017
Albumin (g/dL)°, mean ± SEM	3.14 ± 0.02	4.01 ± 0.02	<0.001
PNI°, mean ± SEM	36.96 ± 0.21	48.23 ± 0.19	<0.001
Creatinine (mg/dL)°, mean ± SEM	1.51 ± 0.11	1.26 ± 0.11	<0.001
eGFR° (mL/min/1.73 m^2^), mean ± SEM	48.8 ± 0.8	51.7 ± 0.7	<0.01
CRP (mg/L)°, mean ± SEM	6.1 ± 0.7	1.2 ± 0.7	<0.001
BB-DNA (ng/mL)°, mean ± SEM	53.7 ± 3.5	42.2 ± 2.9	0.015

AF, atrial fibrillation; BB-DNA, blood bacterial DNA; CKD, chronic kidney disease; COPD, chronic obstructive pulmonary disease; CRP, C-reactive protein; eGFR, estimated glomerular filtration rate; IHD, ischemic heart disease; PNI, Prognostic Nutritional Index; RBC, red blood cells; SD, standard deviation; SEM, standard error of the mean. ° For variables marked with °, *p* values were obtained by ANCOVA adjusted for age and sex.

**Table 2:** Associations of BB-DNA and PNI with mental status alterations and behavioral disturbances.

	Model 1 OR (95% CI) *N* = 1016	Model 2 OR (95% CI) *N* = 1016	Model 3 OR (95% CI) *N* = 1016	Model 4 OR (95% CI) *N* = 1016
ACMS (*N* = 68; 6.7%)				
Log BB-DNA	1.93 (1.14–3.29)	1.75 (1.09–2.81)	1.74 (1.08–2.79)	1.76 (1.09–2.82)
Low-PNI	0.83 (0.50–1.35)	0.94 (0.55–1.59)	0.92 (0.54–1.57)	0.98 (0.55–1.74)
ED (*N* = 413; 40.5%)				
Log BB-DNA	0.95 (0.75–1.20)	0.98 (0.78–1.23)	0.97 (0.77–1.23)	0.96 (0.75–1.23)
Low-PNI	1.70 (1.28–2.24)	1.75 (1.29–2.35)	1.74 (1.29–2.34)	2.24 (1.60–3.13)
EDS (*N* = 98; 9.6%)				
Log BB-DNA	1.41 (0.95–2.09)	1.38 (0.93–2.05)	1.37 (0.92–2.04)	1.37 (0.91–2.08)
Low-PNI	0.67 (0.43–1.04)	0.72 (0.44–1.14)	0.71 (0.44–1.12)	0.79 (0.48–1.32)
MFV (*N* = 278; 27.4%)				
Log BB-DNA	1.13 (0.87–1.46)	1.11 (0.85–1.45)	1.11 (0.86–1.44)	1.11 (0.84–1.45)
Low-PNI	0.83 (0.61–1.12)	0.88 (0.64–1.21)	0.88 (0.63–1.21)	0.95 (0.67–1.34)
BD (*N* = 71; 7.0%)				
Log BB-DNA	3.05 (1.11–8.42)	3.09 (1.11–8.59)	3.08 (1.10–8.57)	3.15 (1.10–9.01)
Low-PNI	1.85 (1.06–3.23)	1.80 (1.05–3.06)	1.78 (1.04–3.04)	1.96 (1.09–3.53)
Cognitive impairment (CPS ≥ 2) (*N* = 466; 45.9%)				
Log BB-DNA	0.93 (0.74–1.17)	0.98 (0.77–1.22)	0.98 (0.78–1.23)	0.96 (0.76–1.20)
Low-PNI	1.42 (1.07–1.89)	1.39 (1.06–1.83)	1.39 (1.05–1.82)	1.42 (1.08–1.88)

Model 1: adjusted for age, sex, number of medications at admission, and estimated glomerular filtration rate (eGFR). Model 2 (primary model): Model 1 additionally adjusted for neutrophil count. Model 3 (sensitivity analysis): Model 2 additionally adjusted for the Charlson Comorbidity Index (CCI). Model 4 (sensitivity analysis): Model 3 additionally adjusted for the Frailty Index. OR, odds ratio; CI, confidence interval; BB-DNA, circulating bacterial DNA; PNI, Prognostic Nutritional Index; ACMS, acute change in mental status from baseline; ED, easily distracted behavior; EDS, episodes of disorganised speech; MFV, mental function varying over the course of the day; BD, behavioral disturbances; CPS, Cognitive Performance Scale.

## Data Availability

The data supporting the findings presented in this article are contained within the manuscript. Additional clinical and biochemical datasets generated or analyzed during the study may be obtained from the corresponding author upon reasonable request, provided that applicable institutional data protection requirements and ethical approvals are satisfied.

## Supplementary Materials

The following supporting information can be downloaded at: https://www.mdpi.com/article/10.3390/nu18203387/s1, Table S1: Comparison of clinical and demographic characteristics between participants with and without available HSV-1/VZV data; Table S2. Associations of circulating bacterial DNA and low PNI with mental status alterations and behavioral disturbances: sensitivity analysis additionally adjusted for infection; Supplementary reporting STROBE checklist.

## Abbreviations

The following abbreviations are used in this manuscript:

ACMS	Acute Change in Mental Status from baseline
BB-DNA	Blood Bacterial DNA
BD	Behavioral Disturbances
CCL22	C-C Motif Chemokine Ligand 22
CCI	Charlson Comorbidity Index
CKD	Chronic Kidney Disease
COPD	Chronic Obstructive Pulmonary Disease
CPS	Cognitive Performance Scale
CRP	C-Reactive Protein
CXCL9	C-X-C Motif Chemokine Ligand 9
DM2	Type 2 Diabetes Mellitus
ED	Easily Distracted
EDS	Episodes of Disorganised Speech
eGFR	Estimated Glomerular Filtration Rate
FGF21	Fibroblast Growth Factor 21
FI	Frailty Index
FST	Follistatin
GDF15	Growth Differentiation Factor 15
HSV-1	Herpes Simplex Virus Type 1
IL-1β	Interleukin-1 Beta
IL-6	Interleukin-6
IL-10	Interleukin-10
IL-17A	Interleukin-17A
LTL	Leukocyte Telomere Length
MFV	Mental Function Varies over the course of the day
NfL	Neurofilament Light Chain
NLR	Neutrophil-to-Lymphocyte Ratio
OPN	Osteopontin
PNI	Prognostic Nutritional Index
TTV	Torque Teno Virus
VZV	Varicella-Zoster Virus

## Appendix A

The flowchart illustrates the selection of study participants and the derivation of the analytical populations. Of the 1108 Report-AGE participants with available biological specimens, 27 were excluded due to in-hospital mortality. Among the remaining 1081 survivors, 65 were excluded because of missing data required for the analyses, resulting in a final analytical cohort of 1016 participants. Additional biomarker assessments were available in subsets of this cohort: HSV-1 and VZV DNAemia were characterized in 456 participants, whereas LTL was assessed in 872 participants. Accordingly, analyses involving HSV-1/VZV and LTL were conducted in these biomarker-specific subsets, whereas analyses involving BB-DNA, TTV, inflammatory biomarkers, and the main clinical variables were based on the full 1016-participant analytical cohort.

BB-DNA, blood bacterial DNA; HSV-1, herpes simplex virus 1; LTL, leukocyte telomere length; PNI, Prognostic Nutritional Index; TTV, Torque Teno Virus; VZV, Varicella-Zoster Virus.

**Table A1:** Inflammatory, metabolic, and neurodegenerative biomarkers across BB-DNA tertiles stratified by immunonutritional status.

Group 1 (Low-PNI)				
	BB-DNA T1 *N* = 150	BB-DNA T2 *N* = 163	BB-DNA T3 *N* = 181	*p* Value
GDF15 (pg/mL)	7981.2 ± 634.8	7939.0 ± 625.0	8452.5 ± 621.0	0.550
NfL (pg/mL)	131.5 ± 20.3	153.2 ± 19.5	141.0 ± 19.9	0.367
CCL22 (pg/mL)	606.93 ± 62.4	608.7 ± 59.8	545.0 ± 61.1	0.267
OPN (ng/mL)	303.0 ± 281.1	293.9 ± 271.0	278.0 ± 281.3	0.417
FST (pg/mL)	2763.4 ± 194.6	2881.9 ± 186.6	2875.4 ± 190.3	0.664
FGF21 (pg/mL)	757.9 ± 178.7	709.8 ± 170.5	784.3 ± 173.9	0.842
**Group 2 (High-PNI)**				
	**BB-DNA T1** ***N*** **= 192**	**BB-DNA T2** ***N*** **= 178**	**BB-DNA T3** ***N*** **= 152**	***p *** **Value**
GDF15 (pg/mL)	4182.2 ± 641.5	4213.1 ± 667.3	4310.5 ± 680.4	0.656
NfL (pg/mL)	85.4 ± 21.9	107.8 ± 22.8	101.4 ± 23.3	0.153
CCL22 (pg/mL)	918.8 ± 62.0	872.2 ± 63.8	873.6 ± 65.8	0.340
OPN (ng/mL)	97.8 ± 13.8	113.9 ± 14.2	111.3 ± 14.7	0.101
FST (pg/mL)	2126.3 ± 195.7	2039.6 ± 201.1	2155.2 ± 207.6	0.589
FGF21 (pg/mL)	434.1 ± 108.4	465.5 ± 111.5	557.7 ± 115.1	0.371

Data are presented as mean ± SEM. *p* values refer to ANCOVA comparisons across BB-DNA tertiles within each immunonutritional status group, adjusted for age, sex, frailty status, infections, CCI, and creatinine levels. BB-DNA, blood bacterial DNA; CCL22, C-C motif chemokine ligand 22; FGF21, fibroblast growth factor 21; FST, follistatin; GDF15, growth differentiation factor 15; NfL, neurofilament light chain; OPN, osteopontin; PNI, Prognostic Nutritional Index; SEM, standard error of the mean; T1, tertile 1; T2, tertile 2; T3, tertile3.

**Table A2:** Associations of PNI and BB-DNA with impairment in specific memory domains at hospital admission.

	Model 1 OR (95% CI) *N* = 1016	Model 2 OR (95% CI) *N* = 1016	Model 3 OR (95% CI) *N* = 1016	Model 4 OR (95% CI) *N* = 1016
Short-term memory (*N* = 429, 42.2%)				
BB-DNA tertiles	1.00 (0.95 –1.05)	0.93 (0.89–1.10)	0.93 (0.79–1.10)	0.92 (0.78–1.10)
Low-PNI	2.02 (1.53–2.67)	1.84 (1.37–2.46)	1.83 (1.36 –2.45)	1.90 (1.42–2.57)
Procedural memory (*N* = 210, 20.7%)				
BB-DNA tertiles	1.02 (0.99–1.04)	1.05 (0.87–1.28)	1.06 (0.87–1.28)	1.08 (0.88–1.32)
Low-PNI	1.12 (0.81–1.55)	1.07 (0.77–1.49)	1.06 (0.76–1.47)	1.25 (0.87–1.79)
Situational memory (*N* = 154, 15.1%)				
BB-DNA tertiles	0.92 (0.79–1.07)	1.03 (0.83–1.28)	1.03 (0.83–1.29)	1.02 (0.81–1.28)
Low-PNI	1.46 (1.01–2.11)	1.44 (1.02–2.15)	1.43 (0.98–2.11)	1.49 (0.99–2.23)

Model 1: adjusted for age, sex, number of medications at admission, and eGFR. Model 2 (primary model): Model 1 additionally adjusted for neutrophil count. Model 3 (sensitivity analysis): Model 2 additionally adjusted for the Charlson Comorbidity Index (CCI). Model 4 (sensitivity analysis): Model 3 additionally adjusted for the Frailty Index. BB-DNA, blood bacterial DNA; CCI, Charlson Comorbidity Index; CI, confidence interval; eGFR, estimated glomerular filtration rate; OR, odds ratio; PNI, Prognostic Nutritional Index.
