# Supplementary material for: Prognostic Nutritional Index, Circulating Bacterial and Viral DNA, and Neurocognitive Vulnerability in Geriatric Inpatients: A Cross-Sectional Study
Source: Nutrients. 2026 Oct 15;18(20):3387. doi: 10.3390/nu18203387 (PMC13708057; doi:10.3390/nu18203387)
Supplement: Supplementary file 1 [file nutrients-18-03387-s001.zip › nutrients-4588466-supplementary.pdf]

Table S1. Comparison of clinical and demographic characteristics between participants with and without available HSV-1/VZV data

	Patients without HSV-1 and VZV DNAemia N = 560	Patients with HSV-1 and VZV DNAemia N = 456	*P* value
**Age (yrs),** mean ± SD	83.0 ± 7.0	82.6 ± 7.4	NS
**Males,** n (%)	257 (45.8%)	215 (47.1%)	NS
**Diabetes,** n (%)	119 (21.2%)	104 (22.8%)	NS
**Hypertension,** n (%)	354 (63.2%)	266 (58.3%)	NS
**AF,** n (%)	120 (21.4%)	76 (16.7%)	0.065
**IHD,** n (%)	81 (14.4%)	56 (12.3%)	NS
**CKD,** n (%)	135 (24.1%)	95 (20.8%)	NS
**COPD,** n (%)	89 (15.9%)	64 (14.0%)	NS
**Cancer,** n (%)	79 (14.1%)	73 (16.0%)	NS
**Infections,** n (%)	132 (23.6%)	73 (16.0%)	< 0.01
**Dementia,** n (%)	95 (16.9%)	89 (19.5%)	NS
**Frailty Index**, n (%)			NS
**< 0.10**	106 (18.9%)	94 (20.6%)	
**0.10-0.19**	209 (37.4%)	165 (36.2%)	
**≥ 0.20**	245 (43.7%)	197 (43.2%)	
**PNI °,** mean ± SEM	44.1 ± 0.3	44.9± 0.4	NS
**Creatinine (mg/dL)°,** mean ± SEM	1.27 ± 0.04	1.17 ± 0.03	NS
**eGFR° (mL/min/1.73 m²),** mean ± SEM	55.7 ± 1.0	57.9 ± 1.0	NS
**CRP (mg/L)°,** mean ± SEM	4.57 ± 0.3	4.48 ± 0.3	NS
**BB-DNA (ng/mL)°,** mean ± SEM	48.8 ± 2.0	44.8 ± 4.2	NS

AF, atrial fibrillation; BB-DNA, blood bacterial DNA; CKD, chronic kidney disease; COPD, chronic obstructive pulmonary disease; CRP, C-reactive protein; eGFR, estimated glomerular filtration rate; IHD, ischemic heart disease; PNI, Prognostic Nutritional Index; SD, standard
deviation; SEM, standard error of the mean.°For variables marked with °, p values were obtained by ANCOVA adjusted for age and sex.

Table S2. Associations of circulating bacterial DNA and low PNI with mental status alterations and behavioral disturbances: sensitivity analysis additionally adjusted for infection.

	Model 1 OR (95% CI) N=1,016	Model 2 OR (95% CI) N=1,016
ACMS (N=68; 6.7%)		
Log BB-DNA	1.61 (1.02–2.61)	1.70 (1.04–2.75)
Low-PNI	0.97 (0.58–1.64)	1.07 (0.61-1.89)
ED (N=413; 40.5%)		
Log BB-DNA	1.03 (0.83-1.29)	1.03 (0.80-1.32)
Low-PNI	1.84 (1.39-2.43)	2.04 (1.47-2.84)

EDS (N=98; 9.6%)		
Log BB-DNA	1.31 (0.89-1.91)	1.33 (0.87-2.03)
Low-PNI	0.69 (0.44-1.08)	0.86 (0.53-1.39)
MFV (N=278; 27.4%)		
Log BB-DNA	1.17 (0.91-1.50)	1.13 (0.85-1.49)
Low-PNI	0.84 (0.62-1.13)	0.90 (0.54-1.49)
BD (N=71; 7.0%)		
Log BB-DNA	3.33 (1.20-9.27)	3.16 (1.07-9.04)
Low-PNI	1.85 (1.09-3.16)	1.87 (1.07-3.28)
Cognitive impairment (CPS ≥2) (N=466; 45.9%)		
Log BB-DNA	0.95 (0.75–1.18)	0.95 0.73-1.23)
Low-PNI	1.44 (1.10–1.89)	1.49 (1.08-2.05)

Model 1: adjusted for age, sex. Model 2 (sensitivity analysis): Model 1 additionally adjusted for number of medications, estimated glomerular filtration rate (eGFR), neutrophil count, the Charlson Comorbidity Index (CCI), Frailty and infection. OR, odds ratio; CI, confidence interval; BB-DNA, circulating bacterial DNA; PNI, Prognostic Nutritional Index; ACMS, acute change in mental status from baseline; ED, easily distracted behaviour; EDS, episodes of disorganised speech; MFV, mental function varying over the course of the day; BD, behavioural disturbances; CPS, Cognitive Performance Scale.

STROBE Statement—Checklist of items that should be included in reports of ***cross-sectional studies***

	Item No	Recommendation	Section/page
**Title and abstract**	1	(*a*) Indicate the study's design with a commonly used term in the title or the abstract	Title, page 1, Line 46 (Abstract)
		(*b*) Provide in the abstract an informative and balanced summary of what was done and what was found	The abstract summarizes the study rationale, objective, design, population, methods, main results, and conclusions, with an appropriately balanced interpretation of the findings.
**Introduction**			
Background/rationale	2	Explain the scientific background and rationale for the investigation being reported	Introduction p. Lines 75-113
Objectives	3	State specific objectives, including any prespecified hypotheses	Lines 114-125
**Methods**			
Study design	4	Present key elements of study design early in the paper	Methods, Section 2.1, p. 3-4
Setting	5	Describe the setting, locations, and relevant dates, including periods of recruitment, exposure, follow-up, and data collection	Methods, Section 2.1, p. 3
Participants	6	(*a*) Give the eligibility criteria, and the sources and methods of selection of participants	Methods, Section 2.1, p. 3,4
Variables	7	Clearly define all outcomes, exposures, predictors, potential confounders, and effect modifiers. Give diagnostic criteria, if applicable	Methods, Sections 2.1, 2.6, p. 4, 9
Data sources/ measurement	8*	For each variable of interest, give sources of data and details of methods of assessment (measurement). Describe comparability of assessment methods if there is more than one group	Methods, Sections 2.1–2.5, p. 4–9
Bias	9	Describe any efforts to address potential sources of bias	Methods, Sections 2.1 and 2.6, p. 4 and 9–10; Discussion, p. 20–21. Strategies to minimize potential bias included the application of predefined selection criteria, standardized procedures, blood sample collection within 24 hours of admission, standardized clinical assessments, adjustment for potential confounders, and sensitivity analyses. Potential residual sources of bias are discussed in the Limitations section.
Study size	10	Explain how the study size was arrived at	Methods, Section 2.1, p. 3-4

			Sample size was determined by the availability of biological samples and clinical and biomarker data from the Report-AGE cohort.
Quantitative variables	11	Explain how quantitative variables were handled in the analyses. If applicable, describe which groupings were chosen and why	Methods, Sections 2.1 and 2.6, p. 4; 7–9 (PNI categorization, BB-DNA tertiles, log transformed data.)
Statistical methods	12	(*a*) Describe all statistical methods, including those used to control for confounding	Methods, Sections 2.6, p. 7–9
		(*b*) Describe any methods used to examine subgroups and interactions	Methods, Sections 2.6, p. 7–9
		(*c*) Explain how missing data were addressed	Methods, Section 2.1, p. 3-4; Statistical Analysis, Figure A1. Participants with missing data required for the respective analyses were excluded from those analyses; the number of participants included in each analysis is reported in the corresponding tables and figures.
		(*d*) If applicable, describe analytical methods taking account of sampling strategy	Not applicable; no complex sampling strategy was used.
		(*e*) Describe any sensitivity analyses	Methods, Sections 2.6, p.8, Table 2, Table A2
**Results**			
Participants	13*	(a) Report numbers of individuals at each stage of study—eg numbers potentially eligible, examined for eligibility, confirmed eligible, included in the study, completing follow-up, and analysed	Results, Sections 3.1–3.4, p. 10–17; Methods, p. 5 (Figure A1)
		(b) Give reasons for non-participation at each stage	Methods, Section 2.1, p. 4; Figure A1
		(c) Consider use of a flow diagram	Figure A1, Appendix
Descriptive data	14*	(a) Give characteristics of study participants (eg demographic, clinical, social) and information on exposures and potential confounders	Results, Section 3.1, p. 9,10; Table 1
		(b) Indicate number of participants with missing data for each variable of interest	Methods, Section 2.1, p. 4; Results, Section 3.3, p. 12; Supplementary Table S1
Outcome data	15*	Report numbers of outcome events or summary measures	Results, Sections 3.1–3.4, p. 9–14; Tables 1–2; Supplementary Table A2, Figures 1–4
Main results	16	(*a*) Give unadjusted estimates and, if applicable, confounder-adjusted estimates and their precision (eg, 95% confidence interval). Make clear which	Results, Table 2; Supplementary Table A2. Adjusted estimates are reported

		confounders were adjusted for and why they were included	with 95% confidence intervals. The variables included in the multivariable models and the rationale for their inclusion are described in Methods, Section 2.6.
		(*b*) Report category boundaries when continuous variables were categorized	Methods, Section 2.1 (PNI), p. 4; Results, Section 3.3, p. 12-13 (BB-DNA tertiles)
		(*c*) If relevant, consider translating estimates of relative risk into absolute risk for a meaningful time period	Not applicable (cross-sectional study; no time-to-event or relative risk estimates requiring translation into absolute risk).
Other analyses	17	Report other analyses done—eg analyses of subgroups and interactions, and sensitivity analyses	Results Section 3.3, 3.4, p. 10-14, Table 2, Table A2
**Discussion**			
Key results	18	Summarise key results with reference to study objectives	Results, p. 10-14; Discussion, p. 15-19; Conclusions, p. 19. The main findings are summarised in relation to the prespecified study objectives, distinguishing the primary associations from secondary and exploratory findings.
Limitations	19	Discuss limitations of the study, taking into account sources of potential bias or imprecision. Discuss both direction and magnitude of any potential bias	Discussion, p.18 and 19
Interpretation	20	Give a cautious overall interpretation of results considering objectives, limitations, multiplicity of analyses, results from similar studies, and other relevant evidence	Discussion, pp. 15–19; Conclusions, p. 19. The findings are interpreted cautiously in relation to the study objectives, cross-sectional design, potential residual confounding, limitations of PNI and BB-DNA as indirect markers, single-time-point assessment of viral DNAemia, and the exclusion of patients who died during hospitalization. The exploratory nature and multiplicity of some analyses are also acknowledged, and the findings are discussed in relation to the available literature.
Generalisability	21	Discuss the generalisability (external validity) of the study results	Discussion, p. 19

Other information			
Funding	22	Give the source of funding and the role of the funders for the present study and, if applicable, for the original study on which the present article is based	Funding section p. 20

*Give information separately for exposed and unexposed groups.

Note: An Explanation and Elaboration article discusses each checklist item and gives methodological background and published examples of transparent reporting. The STROBE checklist is best used in conjunction with this article (freely available on the Web sites of PLoS Medicine at http://www.plosmedicine.org/, Annals of Internal Medicine at http://www.annals.org/, and Epidemiology at http://www.epidem.com/). Information on the STROBE Initiative is available at www.strobe-statement.org.
